# Mechanical and Tribological Properties of 3D Printed Polyamide 12 and SiC/PA12 Composite by Selective Laser Sintering

**DOI:** 10.3390/polym14112167

**Published:** 2022-05-27

**Authors:** Guoyan Yu, Jingdong Ma, Jun Li, Jingquan Wu, Jiang Yu, Xianzhang Wang

**Affiliations:** 1 School of Mechanical and Power Engineering, Guangdong Ocean University, Zhanjiang 524088, China; yugy@gdou.edu.cn (G.Y.); majingdong2022@126.com (J.M.); uu337@163.com (J.L.); lengfeng402@163.com (J.W.); gdouyj@163.com (J.Y.); 2 Guangdong Provincial Marine Equipment and Manufacturing Engineering Technology Research Center, Zhanjiang 524088, China

**Keywords:** polymeric composites, selective laser sintering, friction, wear mechanism

## Abstract

Polymeric matrix composites are important to the advancement of industries such as the automobile and medicine industries. In this study, the silicon carbide (SiC) particle-reinforced polyamide12 (PA12) matrix composites were fabricated by selective laser sintering system as well as the pure PA12. The surface topographies, mechanical, and tribological properties were further examined. The results indicated that the friction and wear resistance of the composite were improved compared with the PA12 matrix. The compressive strength increased about 8.5%, shore D hardness increased about 6%. The friction coefficient decreased about 10%, the specific wear rate decreased 20% after adding silicon carbide 10% weight to PA12. The wear mechanisms were also discussed. The deformed asperities on the worn surface can withstand more tangential load, and therefore resulted in lower specific wear rate. It was found that the content of SiC particles on the surface were reduced after friction tests. According to the analysis of SEM, EDS, and FTIR results, the wear mechanisms were considered to be the abrasive and fatigue mode. This type of PA12 matrix composite might be a promising potential in marine and energy applications.

## 1. Introduction

Additive manufacturing (AM), commonly known as 3D printing, is a technique that creates objects based on digital models through a layer-by-layer accumulation approach. Selective laser sintering (SLS) is a type of industrial powder bed fusion (PBF) process for the fabrication of polymeric components, which has been widely used in the preparation of polymer and its composites. It employs a CO_2_ laser to provide sufficient thermal energy to scan and sinter each cross-section of targeted objects to fabricate polymeric products. Polyamide 12 (PA12) has good mechanical properties, low relative density, low water absorption and melting point, and low quality due to its chemical structure characteristic [1]. It has become the most widely used polymer in the SLS system owing to its low processing temperatures, low laser power requirement, and high accuracy [2,3].

Hence, extensive research has been conducted on the chemical, physical, and mechanical properties of PA12 parts printed by using SLS. The effect of the energy density [4] and build orientation [5] of SLS-printed PA12 specimens was investigated. Dadbakhsh et al. [6] investigated the effect of raw and used PA12 powder on the coalescence fusing behavior, crystallinity, microstructure, and mechanical properties. Mehdipour et al. [7] investigated the orientation and rate dependent mechanical behaviors under tensile tests. Balemans et al. [8] developed a numerical model based on the finite element method to solve the flow, temperature, and crystallization kinetics of PA12 powder in SLS. Yang and Chen [9] proposed a kinetic model for SLS degradation of PA12, which considers laser effects, sample degradation rates, and oxidation time. Sindinger et al. [10] investigated the effect of build orientation, wall thickness, and porosity characteristics on the mechanical properties. Cai et al. [11] researched the physicochemical characterization of raw powder materials and their printed specimens, as well as the mechanical performance and printing characteristics of printed objects. PA12 was used to fabricate the specimen/scaffold and demonstrate its biocompatibility for scaffold applications. [12]. Wörz et al. [13] identified the influence of the building orientation on the tribological properties. Bai et al. [14] studied the effect of surface orientation on the wear properties and found that the wear resistance was greater and the coefficient of friction was smaller for the side surfaces as compared to the top surfaces. Polymeric matrix composites (PMCs) reinforced with one or more reinforcements can achieve tailored properties more efficiently. They show outstanding physical and chemical properties, including high modulus, high strength, strong designability, good fatigue performance, chemical corrosion resistance, etc. Zaghloul et al. [15] carried out a comprehensive review on the influence reinforcing fibers play on wear behavior of PMCs. The applied load, fiber length, fiber volume fraction, coefficient of friction, and chemical treatment of fibers were analyzed with respect to wear performance of PMCs. The printed PA12 matrix composites prepared by the SLS have increasingly been attracting more and more researchers’ attention [16]. Cano et al. [17] investigated the effect of temperature on the fracture behavior of PA 12 and glass-filled PA 12. Hao et al. [18] found that printed PA12 composites with glass fiber-reinforcements have a higher tensile strength and elastic modulus. Similarly, carbon fibers were used to reinforce PA12 powders for producing additively manufactured composites in Eichenhofer et al.’s study [19,20]. The addition of carbon fiber to the PA12 resulted in increases of the strength and modulus of the plastic, although the strain-to-failure was reduced [21]. The surfaces of carbon fibers were modified with nitric acid to increase the interfacial adhesion between the fiber and PA12 matrix in SLS powders. This approach resulted in an increase in the tensile strength and modulus and a decrease in the sample porosity [22].

A variety of nanomaterial was used as the reinforcements to improve the material properties. Zaghloul et al. [23] reviewed natural fibers and nano fillers improving the performance of polymeric composite materials and prospected the future developments of natural fibers and nano fillers reinforced polymeric composites in the future. Zaghloul et al. [24] investigated the fatigue and tensile behaviors of fiber-reinforced thermosetting composites embedded with nanoparticles. They found 4% weight percentage of CNC could lead to the highest tensile and fatigue strengths of glass fiber-reinforced polyester (GFRP) composites. The fabricated carbon nanotube (CNT)/Polymer composite exhibits significant improvements in the electrical conductivity [25] and thermal conductivity [26] up to anti-static and conductive range. Similarly, Bai et al. [27] found that the elastic modulus was dramatically enhanced, while a decent dispersion of CNT was observed due to the shear thinning effect. Salmoria et al. [28] evaluated the structural and mechanical properties of the composite (PA/MWCNTs). They found higher values of flexural modulus and ultimate strength, and the viscoelastic properties were also improved with the addition of MWCNTs due to the intermolecular interaction between the PA12 and MWCNTs. In addition, the strength was also improved and creep failure was retarded. Zaghloul et al. [29] found that the graphite and MWCNT fillers can lead to a remarkable increase in the flexural and tensile modulus of polypropylene composites. The electrical conductivity monotonously increased with the increase of the weight percentage of the filler when it was less than 8% weight fraction. Zhu et al. [30] composited graphene nanoplatelets (GNP) with PA12 and increase in elastic modulus, but the elongation at break dropped substantially. Berti et al. [31] investigated the main mechanical characteristics of the material and the effect of the manufacturing anisotropy on the mechanical performances of PA/Al2O3 composite. Zaghloul et al. [32] investigated the influence of flame-retardant magnesium hydroxide on the mechanical properties of high-density polyethylene composites, then Zaghloul [33] investigated the mechanical properties of linear low-density polyethylene fire-retarded with melamine polyphosphate. Bai et al. [34] found that by incorporating the MoS2 filler into the PA12 matrix, the impact property was improved and the friction coefficient and wear rate were reduced significantly. Liu et al. [35] evaluated the effects of nano-sized 58S bioactive glass (nano-58S) on microstructure, mechanical properties, bioactivity, and biocompatibility of the composite scaffolds. They found that the mechanical properties and storage modulus were improved with a small amount of nano-58S. Silicon carbide has the characteristics of high hardness, high wear resistance, high corrosion resistance, and high temperature strength. However, very little work has been done on the surface characteristics and tribological properties of polymer matrix composites reinforced with SiC particles. In this study, the physicochemical characterization and tribological performances of PA12 and SiC/PA12 composite specimens were investigated. This work might provide a reference to the potential application of PA 12 and its composites in industrial fields.

## 2. Materials and Methods

### 2.1. Materials and Sample Preparation

The near-spherical PA12 powders 3300PA, (Hunan Huashu Hi-tech, Changsha, China), with an average particle size of around 20 μm, were chosen as the laser sintered matrix material, and micron SiC with mass fraction of 10% as reinforcement material was added to PA12 matrix. The physical and chemical properties of PA12 and SiC are shown in Table A1 and Table A2 in Appendix A. The PA12 powders pre-incorporated micron SiC particles via mechanical mixing before they were used for laser sintering to ensure a uniform distribution of feedstock. The mixing time was 3 h in a blender at a temperature of 25 °C, and the rotating speed was 300 rpm. A mixture of PA12 and SiC was placed in the powder feeding cylinder of selective laser sintering equipment (pFormics, Hunan Huashu Hi-tech, Changsha, China). The specific sintering process is as follows: the mixtures were firstly preheated at 170 °C and 5% oxygen content for one hour. Next, the SiC/PA12 composite samples were constructed under the conditions of set scanning speed (3000 mm/s), laser power (10 W), laser scanning spacing (0.25 mm), and single-layer powder thickness (0.2 mm) for one hour. After naturally cooling at room temperature for 12 h, SiC/PA12 composite specimens with a size of 30 mm × 30 mm × 5 mm were obtained for the tribological tests. The shape of the specimens for the compression test was solid cylinder, the length was 15 mm, and the diameter was 10 mm. The preparation process of PA12 samples is the same as that of the SiC/PA12 composite.

### 2.2. Mechnical Test

An electronic universal testing machine (Guangzhouweiyi WDW100, Load Cell: 100KN, Guangzhou, China) was used to carry out compression testing of specimens and in accordance with GB/T 1041-92 [36], which is a Chinese version of ISO 604 for determining the compression properties of materials. In the tests, five compression specimens of each type of prepared samples were examined and the average values of compressive strengths were recorded. The hardness of the PA12 and SiC/PA12 composites are measured on LX-D Pointer type Shore hardness tester at a load of 50 N with a 10 s dwell times. Five points for each sample are measured to obtain an average value.

### 2.3. Friction and Wear Performance Test

The tribological measurements of SiC/PA12 composite and PA12 were performed on a Universal Micro-Tribotester (UMT-3, Bruker, Billerica, MA, USA) with a rotating ball-on-disk mode to obtain the friction coefficient. The 304 steel balls with 8 mm-diameter were selected as solid material sliding against the specimens. The applied normal load was chosen as 20 N, and a revolution speed of 360 rpm was employed. During the sliding process, the normal force and friction force were measured and recorded, and the friction coefficient value was calculated automatically. All the tribological experiments were carried out at room temperature. Subsequently, each specimen was weighed for five times, and then the average value was calculated. The wear volume and topography of wear tracks were measured using a three-dimensional (3D) surface profiler (Contour GT-K, Bruker, Billerica, MA, USA). The following two equations are used to calculate the wear rate (*K*):

(1) $$K=\frac{W_{v}}{PS}$$

where $W_{v}$ is the wear volume, it can be obtained by integrating the cross-sectional area of the surface morphology at the wear mark, $m^{3}$. *P* is normal load, N; *S* is the sliding distance, m, it can be calculated from equation:

(2) S=2πr·n·t

where *r* is the radius of rotation, *n* is the speed, *t* is the sliding time.

### 2.4. Characteristics

The 3D topography was measured by a Contour GT-K three-dimensional (3D) surface profiler (Bruker, Billerica, MA, USA). The surface and the cross-section morphology, as well as the wear tracks of the PA12 and SiC/PA12, were examined by a S4800 scanning electron microscope (SEM). FTIR analysis is a significant tool to understand the interaction between the matrix and the fillers. The molecular structure of the sample surface and wear tracks was characterized by Fourier transfer infrared spectroscopy (FT-IR) spectra, which was recorded by a Thermo Scientific Nicolet IS5 (Waltham, MA, USA) between 550 and 4000 cm^−1^. FTIR and SEM equipped with an energy dispersion spectrometer (EDS) were applied to clarify and investigate the wear mechanisms. The EDS linear scan analysis method that the distribution curves of changes in element content are obtained by uses the electron beam scan along with an analytical line. By comparing the EDS and SEM image analysis results of sample, the distribution of elements in different regions can be intuitively obtained.

## 3. Result and Discussion

### 3.1. Characterization of Surfaces

**Figure 1:**
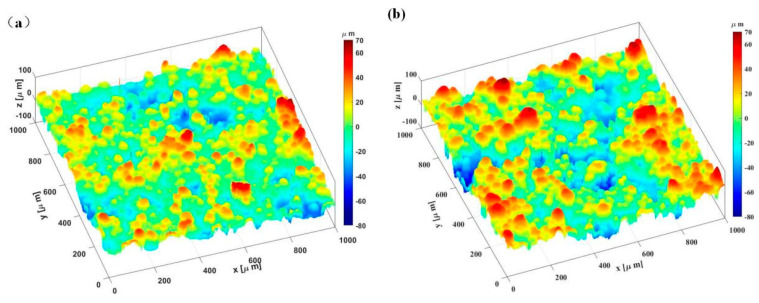
Three−dimensional morphologies of specimen surfaces (**a**) pure PA12 (**b**) SiC/PA12 composites.

The surface topography of the PA12 and SiC/PA12 composites are shown in Figure 1. These surfaces look like “Greenwood and Williamson” surfaces [37], which have been investigated by numerous researchers [38,39,40]. They also look like hemispheres arranged on the surfaces that have been widely studied [41,42,43], most of which considered metallic materials. Different from the regular rough surfaces processed by traditional methods, numerous powders are arranged in a random array on the rough surface processed by selective laser sintering (SLS). As can be seen from Figure 1b, these two materials showed similar roughness trend, and the SiC/PA12 composites showed a higher roughness than that of PA12. The calculated Root Mean Square (RMS) increased by about 49%. The displayed asperities are nearly spherical. Generally, spherical powders are of good flowability and therefore are conducive to obtaining smooth layers and higher packing densities.

**Figure 2:**
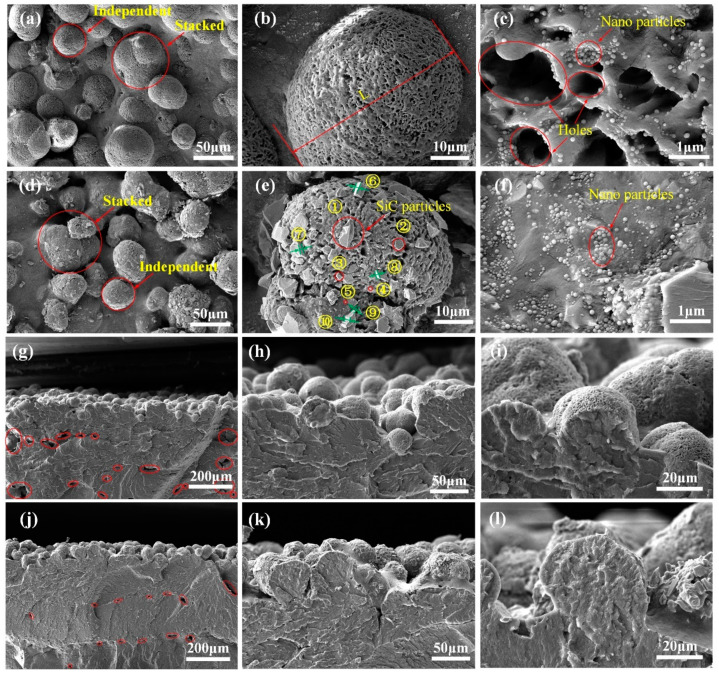
SEM images of the printed specimens. (**a**–**c**) are the surface images of the pure PA12 at magnifications of 1000, 5000, and 50,000, respectively. (**d**–**f**) are the surface images of the SiC/PA12 composites at magnifications of 1000, 5000, and 50,000, respectively. (**g**–**i**) are the cross-section images of the pure PA12 composites at magnifications of 300, 1000, and 3000, respectively. (**j**–**l**) are the cross-section images of the SiC/PA12 composites at magnifications of 300, 1000, and 3000, respectively.

Figure 2 shows the surface and cross-sectional scanning electron microscopy (SEM) images of the PA12 and SiC/PA12 composites with different magnifications. These images confirmed the microstructure of all the specimens shown in Figure 1. The particles on the surface emerged aggregation phenomenon in local regions to form asperities. PA12 powders showed near-spherical morphologies, with the diameters about 10–30 μm distributed on the surface of PA12 and SiC/PA12 composites in the form of superposition or independence (Figure 2a,d). Figure 2b,e shows the high magnification images of the red square area in Figure 2a,d. The size of PA12 powder is also measured, as shown in Figure 2b, and the length of the single powder in the figure is about 49.4 μm. The SiC particles at different locations are numbered from 1 to 10 in Figure 2e. From Number 1 to number 5, the diameters are measured, the maximum diameter is 7.25 μm, and the minimum one is 0.6 μm. From Number 6 to number 10, the thickness was estimated. It has a little change, the average value is 0.82 μm, and the RMS error is 0.27 μm. Numerous micropores are connected to the surface of PA12 powders, the surfaces of which are adhered to numbers of SiC particles for SiC/PA12 composites. The SEM micrographs of PA12 and SiC/PA12 composite with a larger magnification are depicted in Figure 2c,f. Numerous nano-particles are adhered to the holes of asperities surface of PA12 and SiC/PA12 composites. Nano-particles are added to power particles to adjust powder melting and forming characteristics. Figure 2g–l shows that the fractured surfaces of PA12 and SiC/PA12 composites are compact and smooth. It seems that the additions of SiC particles can achieve more effective densification in the PA12 matrix. As can be seen from Figure 2g,j, the cross section of SiC/PA12 composite is fully dense, and only a few pores exist. It indicated that the addition of SiC particles reduced the size and number of pores and increased the densification of the printed specimen.

### 3.2. Mechanical Properties

**Figure 3:**
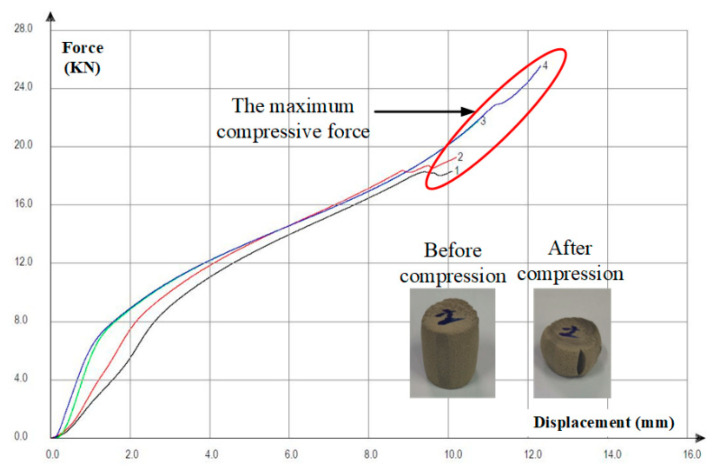
Variation curve of compressive force of SiC/PA12 composites.

Figure 3 depicts the variation curve of compressive force of SiC/PA12 composites during the mechanical tests. As can be seen from Figure 2, the crack is almost parallel to the axis of the cylindrical specimen. The calculated t compressive strength of the composites is 119.42 Mpa, and that of the matrix is 110 Mpa. It clearly shows that the SiC/PA12 composites exhibits an improved compressive strength compared with the pure PA12.

**Figure 4:**
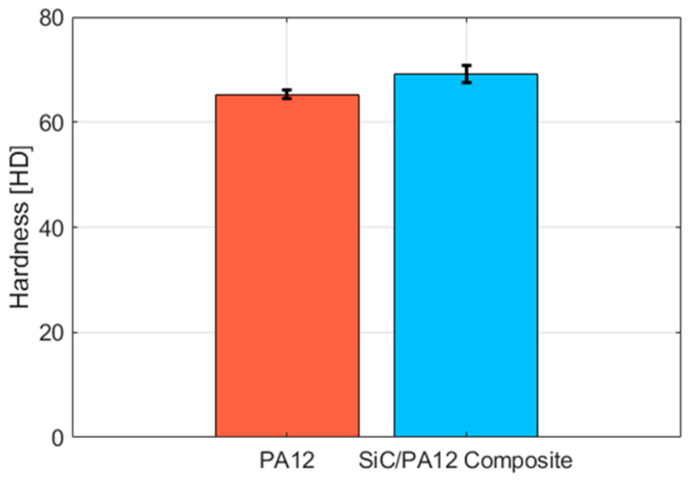
The shore D hardness of pure PA12 and SiC/PA12 composites.

Figure 4 shows the shore D hardness of pure PA12 and SiC/PA12 composites, the values are 65.3 and 69.2 on shore D scale, respectively. The hardness of SiC/PA12 composites increased by about 6% due to the addition of SiC particles.

### 3.3. Tribological Properties

**Figure 5:**
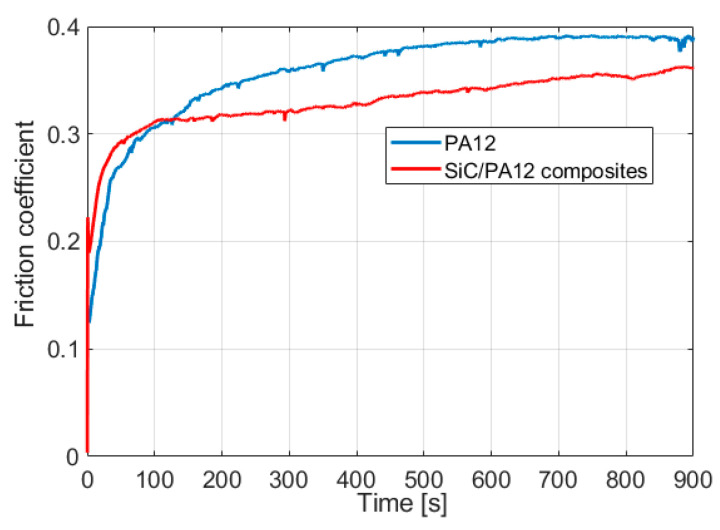
Evolution of the friction coefficient during the sliding tests.

The friction coefficient of PA12 and SiC/PA12 composites are shown in Figure 5. The friction process can be divided into two stages. In the first stage, the friction coefficient of both samples increases dramatically for about 120 s. The friction coefficient of SiC/PA12 is lower than that of PA12. Then, the friction coefficients enter into the second stage and display a slow increase to a steady state. It is observed that the friction coefficient of SiC/PA12 composites is less than that of PA12 around 10%.

**Figure 6:**
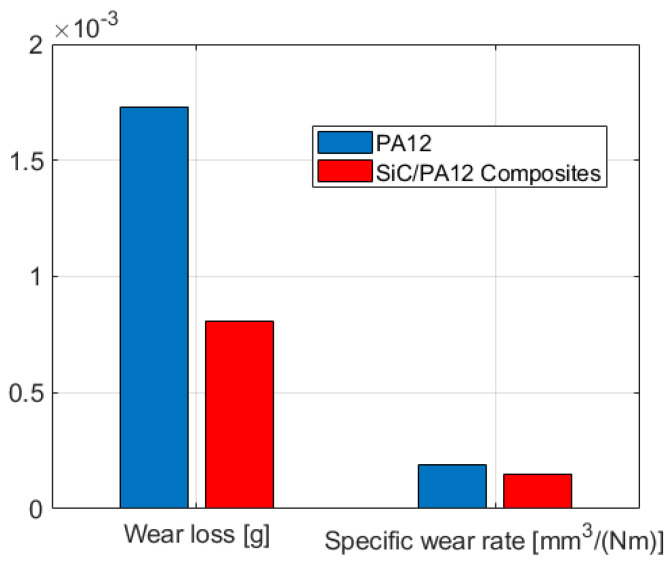
The wear loss and specific wear rate of PA12 and PA12/SiC composite.

**Figure A1:**
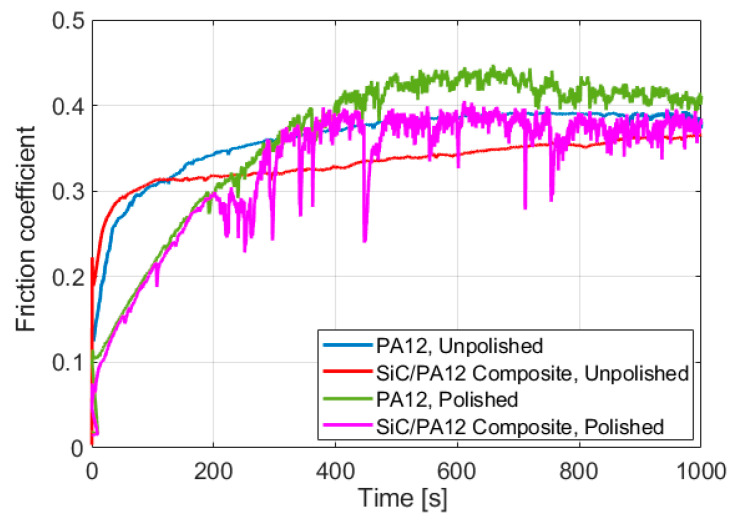
Friction coefficient of PA12 and SiC/PA12 composites for unpolished and polished cases.

The wear losses and wear rates of the PA12 and SiC/PA12 composite are shown in Figure 6. The wear loss of the PA12 and SiC/PA12 composite are 1.73 mg and 0.8 mg, respectively. It is shown that the wear loss of SiC/PA12 composites is about half that of PA12. According to Equations (1) and (2), the corresponding wear rate is 1.88 × 10^−4^ mm^3^/(N·m) and 1.5 × 10^−4^ mm^3^/(N·m), respectively. SiC/PA12 composites exhibited a lower wear loss and specific wear rate than that of PA12. The wear loss decreased 53%, and the specific wear rate decreased about 20%. The results indicated that the wear resistant of the PA12 composites could be enhanced with the addition of SiC particles. They can withstand more normal and tangential forces because of the increase of the hardness and strength of the asperities and the base, which is already illustrated in Figure A1 in Appendix B.

**Figure 7:**
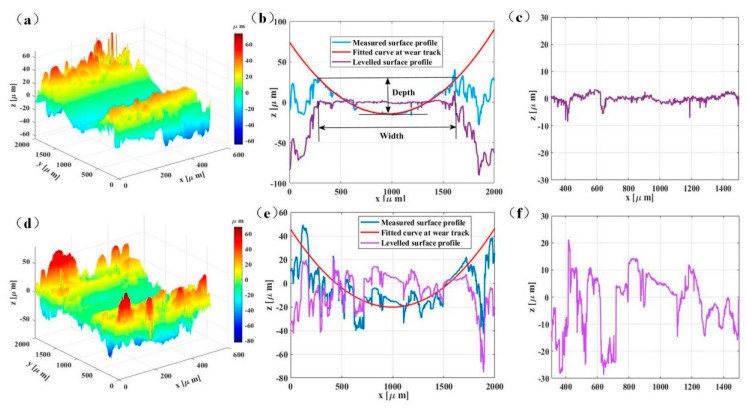
Three−dimensional morphologies (**a**,**d**), cross−sectional profiles (**b**,**e**), and leveled curves (**c**,**f**) of wear tracks of pure PA12 and SiC/PA12 composite.

Figure 7 shows the worn three−dimensional topographies and fitted curves of wear tracks of PA12 and SiC/PA12 composite. The 3D topographies of the wear tracks of PA12 and PA12/SiC composite after the sliding wear tests are shown in Figure 5. The wear tracks on the surface of PA12 and SiC/PA12 composites sample are different. As shown in Figure 7a,d, the wear track of the PA12 is smoother and deeper than that of the SiC/PA12 composites. Two-line profiles of the 3D surfaces are selected to further obtain the fitted curve at wear tracks (Figure 7b,e). Levelled surface profile was obtained by measuring the surface profile minus the fitted curve, as shown in Figure 6. The results clearly show that the wear tracks surface of SiC/PA12 composite are much rougher than that of PA12. In addition, the surface roughness both inside and outside of wear tracks increases due to the addition of SiC particles.

### 3.4. Wear Mechanism Analysis

**Figure 8:**
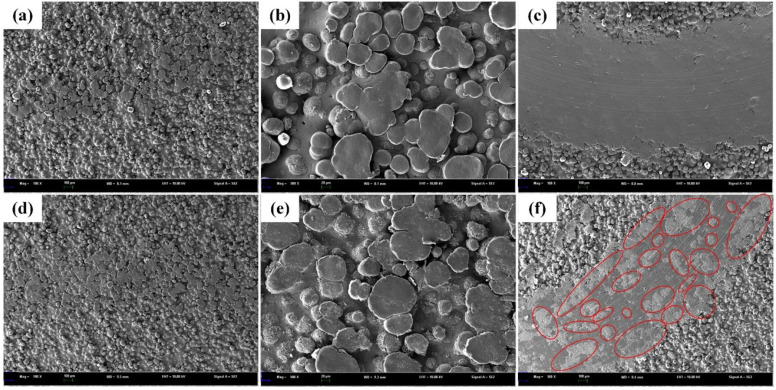
SEM images of wear tracks of PA12 (**a**–**c**) and SiC/PA12 composites (**d**–**f**), with magnifications of 100, 500 and 100.

In order to understand the wear mechanisms of each sample, the morphologies of the wear tracks after a dry sliding wear test were observed and the results are given in Figure 8. After more than 5000 sliding cycles, no microcracks were observed on the worn surfaces of these specimens, suggesting their resistance to plastic deformation. Only a few asperities are in contact and deformed at the initial stage. Meanwhile, a narrow wear track was observed, as shown in Figure 8a,d. Figure 8b,e shows microstructures with a high magnification of the area, and the deformation depends on the height distribution of the asperities. After the process of sliding, the depths and widths become larger due to the combination of the normal and tangential loading. The parallel grooves and scratches along the sliding direction and layer-shaped cracks resulted from abrasive wear, as seen in Figure 8c,f. It suggested that the combined abrasive and fatigue wear is dominant in the wear tests of all samples. As can be seen from Figure 8c,f, there are some deformed asperities on the wear tracks of SiC/PA12 composites surface, while the wear track of PA12 is much smoother. Compared with the cases of the PA12, the number of wear grooves is larger due to the presence of worn SiC particles during sliding. The width of the wear track of SiC/PA12 composites is smaller than that of PA12. Therefore, SiC addition improves the wear resistance of the PA matrix due to the hardness improvement, which is consistent with the results of the friction coefficient and specific wear rate as given in Figure 5 and Figure 6.

**Figure 9:**
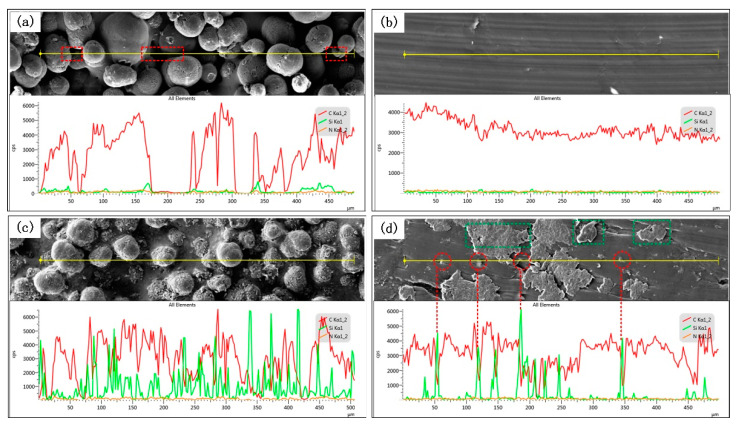
The SEM images and corresponding line scanning EDS analysis. (**a**) Outside of wear tracks for Pure PA 12 specimens. (**b**) Inside of wear tracks for Pure PA 12 specimens. (**c**) Outside of wear tracks for SiC/PA12 composite specimens. (**d**) Inside of wear tracks for SiC/PA12 composite specimens.

Figure 9 shows the microstructures of worn surfaces with a high magnification and corresponding elemental EDS line scan, as well as the quantitative elemental analysis of PA12 and SiC/PA12 composites. Line scans of inside and outside of wear tracks of PA12 and SiC/PA12 composite for Carbon, Silicon, and Nitrogen are presented. The microstructure outside of the wear track of PA12 is shown in Figure 9a. The carbon content mostly from PA12 is higher, except for the locations where the valleys of the asperities are hardly detected. The contents of silicon and nitrogen are somewhat low. The EDS line scan inside of the wear track is shown in Figure 9b and the content of carbon on the scanning line is relatively stability, as the surface is smoother due to the sliding test. The overall content of silicon did not change significantly. The content of silicon is representative of SiC to some extent. Hence, the content of silicon increases due to the addition of SiC particles in SiC/PA12 composite samples, as shown in Figure 9c. Figure 9d shows the microstructure and EDS image of the wear tracks, and the nearly flattened deformed asperities can be seen clearly, as well as the SiC particles (in the red circle). The content of SiC is much lower compared with Figure 9c, which suggests that some of the SiC particles were worn off or buried in the asperities and PA12 matrix. Compared with Figure 9b, the remaining deformed asperities can still sustain some shear force and reduce the real contact area, which might be one of the main reasons that the SiC/PA12 composites have better tribological properties.

**Figure 10:**
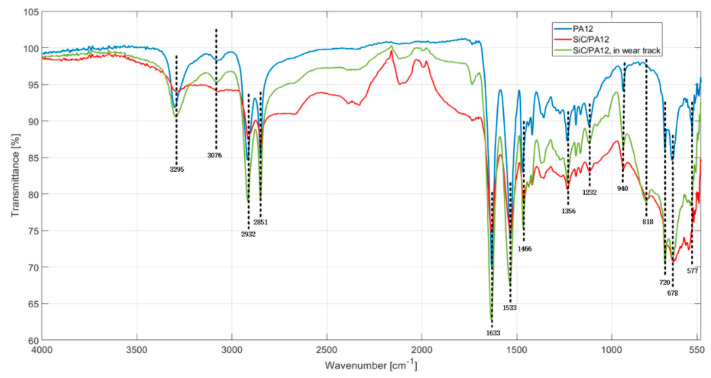
FTIR spectra of the surface and wear track of the sample.

FTIR test is carried out to investigate the chemical components’ inside and outside wear tracks of the PA12 sample and SiC/PA12 composite. The FTIR absorption spectra of the three samples are shown in Figure 10. The blue line, red line, and green line represent the FTIR absorption spectra of PA12, outside and inside of SiC/PA12 composite, respectively. For the PA12 sample, the peaks at 3295 cm^−1^, 3076 cm^−1^, and 1533 cm^−1^ are the stretching vibration adsorption of N-H, the moderate amide B bond, and amide II band (C-N stretching vibration plus CO-NH bending), respectively. The peak at 678 cm^−1^ is the amide V band (N-H out-of-plane bending), and the peak at 577 cm^−1^ is the amide VI band (C-O out-of-plane bending). There are distinct peaks at 2932 cm^−1^, 2851 cm^−1^, and 1633 cm^−1^, corresponding to asymmetric stretching vibration of CH_2_, symmetric stretching vibration of CH_2_, and the stretching vibration of amide C-O, respectively. For the SiC/PA12 sample, in the spectrum of the outside of the wear track, in addition to the functional peaks for the PA12 sample, one more apparent peak appears at the wavenumber of 820 cm^−1^, indicating that a variety of Si-C groups are formed in the sample. However, the characteristic peaks also have some deviation. The amide VI band at 577 cm^−1^ shifts to 592 cm^−1^. The amplitude and width of the moderate amide B bond also have obvious changes. In the spectrum of the outside of the wear track, the results are similar to the one obtained from the outside, which suggests that the integrated structure of the inside case sample is still consisting of SiC particles. The amplitudes of the peak where the wavenumbers are 2932 cm^−1^, 2851 cm^−1^, 1633 cm^−1^, and 1533 cm^−1^ increase significantly, indicating that the absorption capability becomes stronger.

**Figure 11:**
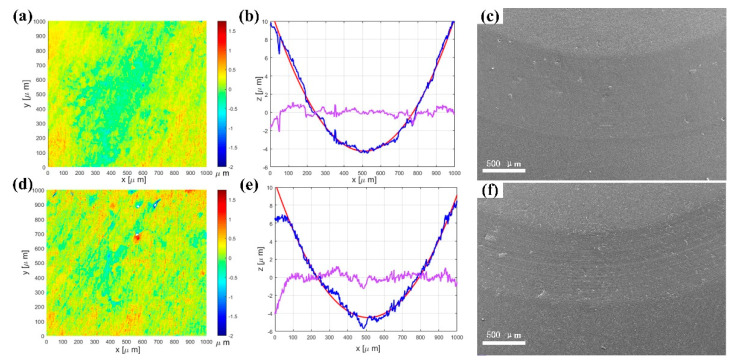
The surface topographies, wear track profiles and SEM images of polished PA 12 (**a**–**c**) and SiC/PA12 composites (**d**–**f**).

The polished samples are used to investigate the potential mechanisms of tribological performances. Surface topographies of the polished pure PA12 and SiC/PA12 composite are shown in Figure 11a,d. The corresponding RMS values are 233.45 nm and 266.93 nm, respectively. The powder size asperities on the surface of polished SiC/PA12 composites are worn off. Next, the friction tests with the same parameters are carried out. The friction coefficients are much higher than that of the original printed surface, as shown in Figure A1. The profiles of selected lines on the wear track of polished samples can be seen from Figure 11b,e. The depth of wear track of SiC/PA12 composites is lower than that of pure PA12, and the leveled curves show that they have close roughness, which is much lower than that in Figure 7c,f. In addition, a similar phenomenon can be seen from Figure 11c,f, which shows the width of the wear tracks of the polished sample is smaller than the original printed surfaces in Figure 7b,d. For the two polished surfaces, the width of the wear tracks of SiC/PA12 composite is smaller than that of PA12, while the micros, cratches are more due to the three-body abrasive wear of SiC particles. Both specimens showed smooth worn surfaces and small amounts of wear particles on the wear tracks. The polished samples can withstand more tangential force because of the larger nominal contact areas.

**Figure 12:**
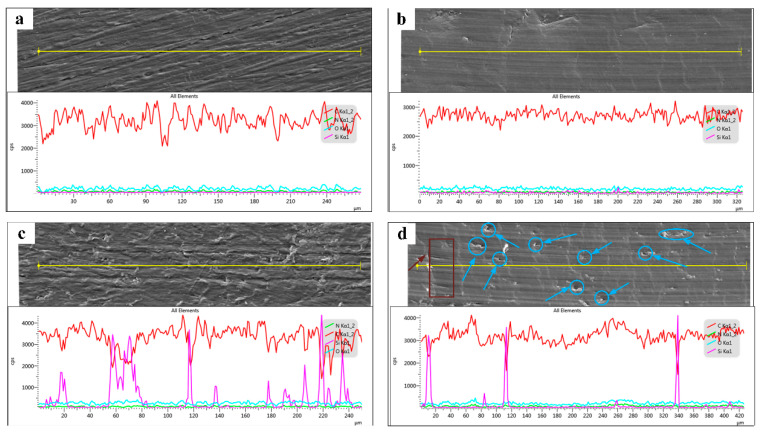
The SEM images and corresponding line scanning EDS analysis for the polished specimens. (**a**) Outside of wear tracks for Pure PA 12 specimens. (**b**) Inside of wear tracks for Pure PA 12 specimens. (**c**) Outside of wear tracks for SiC/PA12 composite specimens. (**d**) Inside of wear tracks for SiC/PA12 composite specimens.

Figure 12 shows the EDS analysis of the inside and outside of wear tracks of PA12 and SiC/PA12 composites. The adhesive and fatigue wear mechanisms still dominate the wear of PA12 and SiC/PA12 composites and it was accompanied by relatively shallow parallel grooves and scratches. It shows some small peaks and valleys on the surface due to the polishing process (see Figure 12a,c). The surface becomes much smoother and the content of carbon only has minor fluctuations around a steady state after friction tests for all the samples (see Figure 12c,d). For the SiC/PA12 composites, the content of silicon only has negligible change, while the content of silicon almost remains the same. This suggests that the SiC particles on the surface are worn off or buried in the PA12 matrix. It also can be seen from Figure 10 that some pits (or holes) were formed on the worn surface of wear track, observed as indicated by arrows. The SiC particles that embedded into PA12 were scraped off by the shear force, and left a variety of pits. This might be a result of the weak bonding force between the SiC particles and PA12 matrix. These released SiC particles led to a third-body abrasion, and therefore resulted in deeper and wider wear grooves of SiC-reinforced PA12 than that of pure PA12.

Above all, the wear tracks of PA12 are deeper and wider than those of SiC/PA12 composites. The asperities of SiC/PA12 composites are not completely flattened or worn off, and they can still sustain tangential force. The real contact area is smaller than that of PA 12, which might be the main reason for the reduction of friction coefficient and wear rate. In brief, according to EDS and wear mechanisms analysis, the main wear mechanisms are abrasive and fatigue modes, and the addition of micron SiC particles in SiC/PA12 composites caused the improvement of mechanical and tribological properties.

## 4. Conclusions

In summary, the SiC/PA12 composites were fabricated by SLS system. Mechanical and tribological properties of the composites were investigated. Moreover, the wear mechanisms were discussed. Some conclusions can be drawn as follows:

(1) The SiC/PA12 composite shows an enhanced compressive strength and hardness with the addition of SiC particles. The compressive strength increased about 8.5%, while the tensile strength did not change too much. The shore D hardness increased about 6%.
(2) The experiment results showed that the surface asperities are near-spherical due to the partly melted PA12 powders. The addition of micron SiC increased the roughness of the PA surface.
(3) The friction coefficient and wear rate of SiC/PA12 composites are improved due to the presence of SiC particles. The friction coefficient reduced about 10%. The wear loss decreased as high as 53%, and the specific wear rate decreased about 20%.
(4) SiC/PA12 composites have better tribological properties because of the combination of the hardness, strength, and deformed powders on the surface. The reduced COF is not only benefitted from the hardness, but also the effect of asperities on the surface.
(5) The wear mechanisms are dominated by abrasive and fatigue wear for both PA12 and SiC/PA12 composite materials, whereas the latter has wear form caused by SiC particles trapped between the surfaces of friction pairs. In addition, the asperities of SiC/PA12 composites have much a higher hardness, which can afford more tangential force.

Thus, the tribological properties of polymers can be tailored by adding hard micro micron and nano particles. It is expected to develop polymeric matrix composites incorporated with multi particles to obtained better mechanical and functional properties, which will be left for future work.

## Appendix A

**Table A1:** The properties of PA12 materials.

Property	Value
Melting point	184 °C
Density	1.02 g$/{\mathrm{cm}}^{3}$
Tensile strength	48 MPa
Tensile modulus	1.82 GPa
Elongation at break	22%
Bending strength	60 Mpa
Bending modulus	1.64 Gpa

**Table A2:** The properties of SiC particles.

Property	Value
Mean grain size	800 nm
Purity	99.9%
Specific surface area	20${\;m}^{2}$/g
Volume density	0.15 g/${\mathrm{cm}}^{3}$
Density	8.9 g/${\mathrm{cm}}^{3}$

## Appendix B

Figure A1 shows the friction coefficient of pure PA12 and SiC/PA12 composites. As can be seen from Figure A1, the friction coefficients of SiC/PA12 composites are lower than of PA material for both unpolished and polished cases. The friction coefficients of polished specimens are higher than that of unpolished cases.
